# Laboratory Evaluation of Storage Stability for Asphalt Binder Modified with Crumb Rubber and Styrene–Isoprene–Styrene Depending on Evaluation Factors and Blending Condition

**DOI:** 10.3390/ma17092091

**Published:** 2024-04-29

**Authors:** Jihyeon Yun, Shyaamkrishnan Vigneswaran, Hyunhwan Kim, Moon-Sup Lee, Soon-Jae Lee

**Affiliations:** 1 Materials Science, Engineering, and Commercialization, Texas State University, San Marcos, TX 78666, USA; yiy1@txstate.edu (J.Y.); gry14@txstate.edu (S.V.); 2 Department of Engineering Technology, Texas State University, San Marcos, TX 78666, USA; k_h82@txstate.edu (H.K.); sl31@txstate.edu (S.-J.L.); 3 Korea Institute of Civil Engineering and Building Technology, Gyeong-si 10223, Republic of Korea

**Keywords:** CRM, SIS, storage stability, SI

## Abstract

Modified asphalt binders are still considered important in asphalt pavement. However, the comprehensive use of various modifiers is limited due to storage stability issues. Moreover, there is a scarcity of detailed analyses regarding the degree of separation for asphalt binders among each method despite the utilization of various methods to assess the storage stability of binders. Therefore, a comprehensive analysis was conducted to assess the storage stability of asphalt binder modified with a crumb rubber modifier (CRM) and styrene–isoprene–styrene (SIS), utilizing five evaluation factors following the ASTM D7173 guidelines based on four mixing methods (A: high-shear mixing method, B: low-speed agitating method, C: high-shear mixing method + low mixing method, D: low-speed agitating method + low mixing method). To produce the modified asphalt binder, the proportions of the CRM were 5% and 10% for each binder, and 10% SIS was added to all binders. The results in this study convey that (1) the addition of the modifier led to an increase in G*/sin *δ* with different mixing methods, but using mixing methods (C and D) for a relatively long time resulted in a lower G*/sin *δ*, indicating suboptimal performance; (2) through the multiple stress creep recovery (MSCR), rheological properties of J_nr_ and % rec exhibited trends similar to G*/sin *δ* evaluation, highlighting an improved elastic recovery with a higher modifier content; (3) storage stability assessment revealed consistent trends in high-shear mixing groups (A and C), while low-speed mixing groups (B and D) exhibited an elevated separation index (SI), suggesting a sensitivity to modification conditions; (4) evaluation using the MSCR method indicated that % rec with a 3.2 kPa load is effective for the sensitive assessment of binder storage stability and J_nr_ showed a limited sensitivity across varying loads, advocating for % rec for precise evaluation; and (5) despite permitting various tests, achieving consistent results remains challenging. Future research should explore diverse modifiers and optimal evaluation methods to enhance knowledge of binder behavior and separation dynamics.

## 1. Introduction

In recent decades, there has been substantial growth and progress in the asphalt industry. In particular, a range of modifiers have greatly enhanced the effectiveness of asphalt binders, addressing numerous issues that arise on road pavement [1,2,3]. Nevertheless, irregular failure for asphalt pavement is on the rise, attributed to factors such as climate change and heightened traffic resulting from population density [4]. In addition, in the case of aged pavement, the deterioration of cracking damage is worsening [5]. Consequently, the significance of selecting an asphalt binder, modifier, and mixture design while taking into account diverse conditions and environments is acknowledged [6,7,8].

Modified asphalt mixtures typically offer multiple benefits such as rutting and crack resistance. However, owing to their heightened viscosity, precise temperature management is essential throughout the production-to-construction process to ensure effective compaction [9,10]. Furthermore, certain modified asphalt binders may incur higher costs, potentially imposing an additional economic drawback on road construction and maintenance, underscoring the significance of comprehensive quality control for both asphalt binders and mixtures [11]. For modified asphalt binders, one of the biggest challenges is ensuring the stability of storage for quality control [12,13,14]. As an example, asphalt binders stored in tanks may undergo a separation between the polymer and bitumen phases. The separation process ultimately degrades the characteristics of the polymer-modified binder and results in the loss of any advantages derived from the modification. Density variations among materials are considered a major factor contributing to this imbalance in storage [15,16,17].

Among the modifiers affecting the storage stability of modified asphalt binders, the crumb rubber modifier (CRM) is considered the most susceptible. This is attributed to the tendency of the CRM to settle due to its higher density compared to asphalt binders [18]. Numerous studies have been undertaken to address and resolve the issues related to storage stability concerning the CRM. In investigations, it was observed that a CRM with smaller particle sizes demonstrated enhanced storage stability performance, and certain studies indicated an improvement in storage stability by incorporating nano materials or a styrene block copolymer [19,20,21,22]. In certain research projects, the storage stability of the binder has been ensured by employing a treated CRM or by subjecting the CRM to a prolonged blending time until it becomes dissolved [23,24]. In addition, improving the storage stability through the application of chemical additives has also been observed in certain instances [25,26]. Therefore, diverse research studies aiming to enhance the storage stability of CRM binders underscore the significance of the thorough assessment of storage stability.

Since the issue of phase separation in modified asphalt binders was raised in the 1980s, numerous methods for evaluating storage stability have been suggested, utilizing a tube, X-ray, microscopy, spectroscopy, etc. [27,28,29,30,31,32]. Regarding tube testing, it has gained widespread recognition and adoption due to its ability to closely simulate the storage conditions of modified binders, making it the most commonly employed method, and various testing procedures are conducted on the final sample [33,34,35,36,37]. It is deemed appropriate to assess the phase separation of modified asphalt binders by examining the storage stability through diverse experimental outcomes. Nevertheless, when appraising phase separation using the binder with various experimental factors, variations in the level of phase separation may occur, leading to ambiguous results for the tester [38].

**Figure 1:**
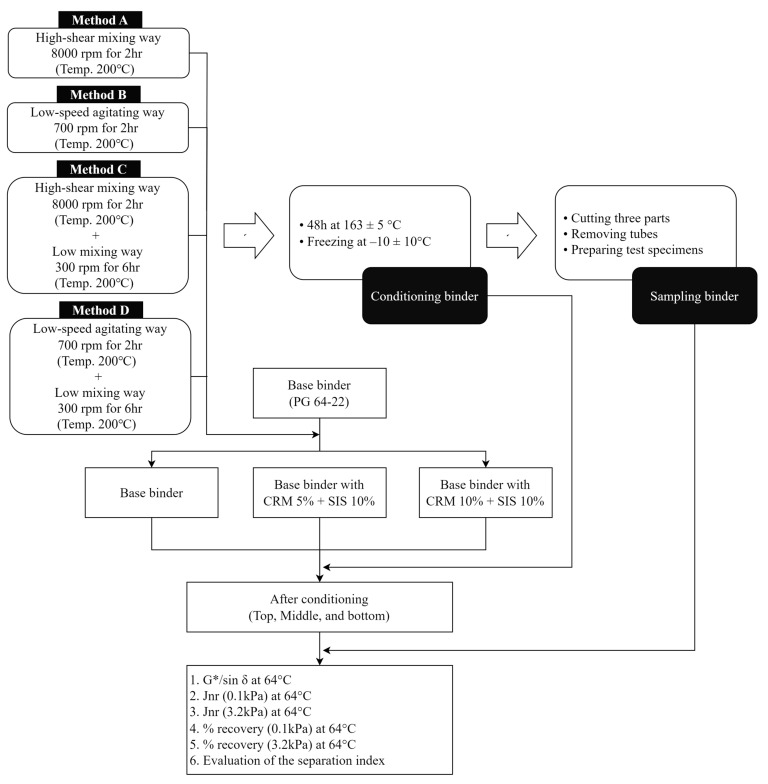
Flow chart of experimental design procedure in this study.

This study, as shown in the flow chart (Figure 1), is based on ASTM D7173 guidelines [39] and utilizes a dynamic shear rheometer (DSR) to examine the variations in storage stability results under the influence of five experimental factors. The modifiers employed include styrene–isoprene–styrene (SIS), known as styrene block copolymers, and a CRM. Four blending methods are introduced to assess how the storage stability is affected. Moreover, suggestions for future research plans and directions will be presented.

## 2. Experimental Design

### 2.1. Materials

**Figure 2:**
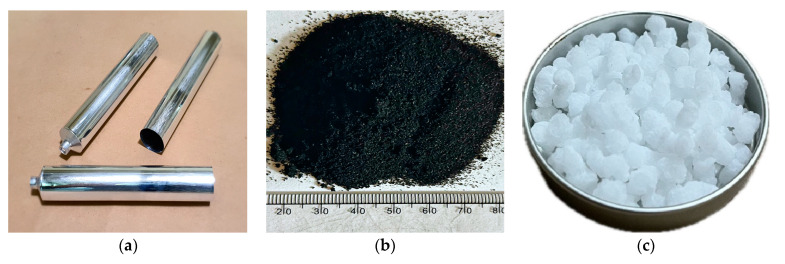
Aluminum tube (**a**), crumb rubber (**b**), and SIS (**c**).

In this research, the base asphalt binder chosen for modification with the CRM and SIS was the PG64-22 asphalt binder (Table 1). The CRM coming from passenger vehicles through the ambient grinding process was selected, and its passing rate is detailed in Table 2. SIS was employed with physical properties similar to those utilized in prior studies, as outlined in Table 3. Aluminum tubes meeting the ASTM D 7173 standard for storage stability evaluation were employed. Figure 2 provides visual representations of each material utilized in this study.

### 2.2. Production and Sampling of CRM+SIS Asphalt Binders

**Figure 3:**
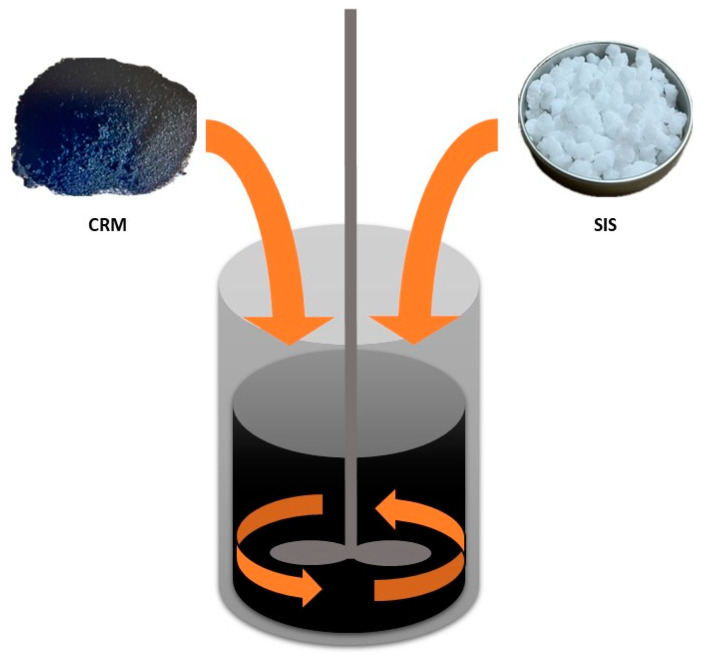
Schematic illustration depicting the mixing method.

In this study, the production of the CRM+SIS-modified asphalt binder involved the sequential addition of a CRM followed by SIS to the base asphalt binder (Figure 3). The proportions of the CRM were 5% and 10% for each binder, and 10% SIS was added to all binders. The blending temperature was set at 200 °C, and the process followed these steps.

- Method A: High-shear mixing (8000 rpm) for 2 h.
- Method B: Low-speed agitating (700 rpm) for 2 h.
- Method C: High-shear mixing (8000 rpm) for 2 h + low mixing (300 rpm) for 6 h.
- Method D: Low-speed agitating (700 rpm) for 2 h + Low mixing (300 rpm) for 6 h.

**Figure 4:**
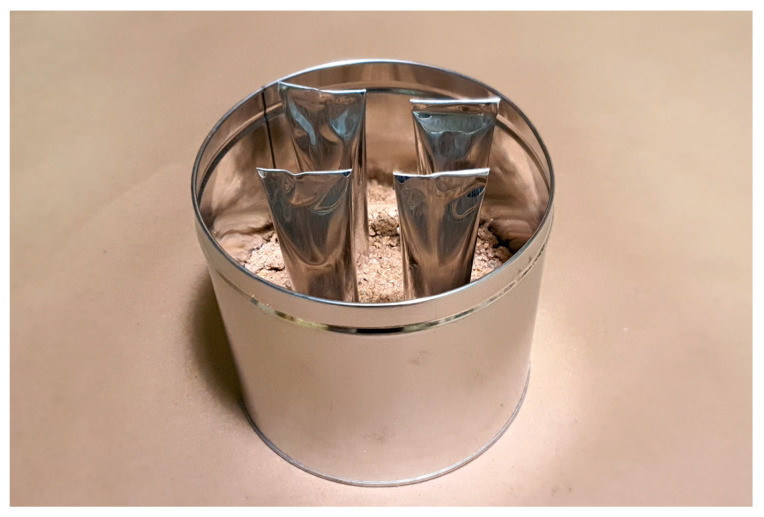
The vertically positioned tube.

Following the blending process, a portion of the CRM + SIS asphalt binder for the original condition sample was promptly set aside for rheological properties. Subsequently, the remaining binder was meticulously mixed and poured into a vertically positioned tube, ensuring an exact mass of 50 ± 0.5 g, before undergoing conditioning in an oven at 163 ± 5 °C for a duration of 48 ± 1 h (Figure 4). Upon completion of this conditioning period, the tubes were transferred to a freezer set at –10 ± 10 °C to achieve thorough solidification of the binder for a minimum of 4 h, maintaining vertical orientation throughout. Once binders had sufficiently hardened, each tube was divided into three nearly equal segments and subjected to a 30 min heating cycle at 163 ± 5 °C to render them fully fluid for the removal of the aluminum tube. Subsequently, binder specimens were meticulously prepared for subsequent evaluations.

### 2.3. Binder Evaluation

#### 2.3.1. Rheological Properties

Due to the dependency of asphalt binder behavior on loading time and temperature, an effective evaluation of asphalt binders should encompass both variables. The DSR serves as an ideal tool for assessing rheological properties, such as the complex shear modulus and phase angle, across medium to high-temperature ranges during asphalt binder testing. By analyzing the viscoelastic behavior, DSR quantifies characteristics such as the complex shear modulus (G*) and phase angle (*δ*) of the asphalt binder. G* reflects the material’s overall resistance to deformation under cyclic shear stress, comprising both elastic (recoverable) and viscous (non-recoverable) elements. On the other hand, δ signifies the proportion of non-recoverable strain. When subjected to a load, asphalt binder exhibits both elastic and viscous deformations. Consequently, through the integration of G* and *δ*, the viscoelastic parameter G*/sin *δ* is calculated at a vibration speed of 10 rad/s. This parameter provides insight into the material’s viscoelastic properties, aiding in the characterization of asphalt binder behavior under varying conditions.

**Figure 5:**
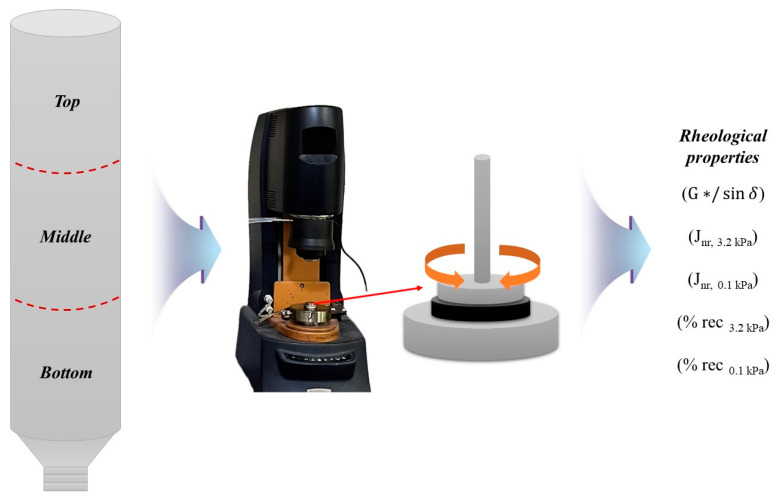
Test procedures for rheological properties.

The multiple stress creep recovery (MSCR) test represents an advancement within the framework of the Superpave asphalt binder specification. One key benefit of the MSCR test lies in its ability to streamline the evaluation process by obviating the necessity for multiple distinct tests such as elastic recovery, toughness, ductility, etc., which are tailored to elucidate polymer deformation within asphalt binders. By consolidating these assessments into a single MSCR test, comprehensive insights into both the performance and composition of the asphalt binder can be gleaned. The methodology of the MSCR test draws upon established principles of creep and recovery testing to assess trends for permanent deformation in a binder. This involves subjecting the asphalt binder sample to a creep load lasting 1 s, followed by a recovery period of 9 s. Initially, a low stress of 0.1 kPa is applied for 10 creep/recovery cycles, which is subsequently augmented to 3.2 kPa for an additional 10 cycles. The MSCR test from traditional PG testing lies in its material response. While PG systems typically measure the high-temperature parameter G*/sin *δ* through the application of oscillatory loads at very low strain rates, the MSCR test employs higher stress and strain levels, thereby providing a more accurate depiction of pavement behavior. However, MSCR and PG systems are both widely utilized to assess asphalt binders. Therefore, both tests were considered in this research (Figure 5).

#### 2.3.2. Separation Index (SI)

This research involved the analysis of a modified asphalt binder, with the SI derived from both top and bottom test outcomes, following the test methods of the ASTM D7173 guidelines. Initially, SI was computed using Equation (1), utilizing the maximum value of G*/sin *δ* between the top and bottom sections, denoted as (G*/sin *δ*)_max_, with the average value from the top and bottom parts represented by (G*/sin *δ*)_avg_. Following this methodology for SI calculation, the SI was assessed using Equations (2) and (3), employing J_nr_ and % recovery for each applied load. Subsequently, a comparative analysis was conducted between the SI results obtained from each source.

(1) $$Separation\;index=\frac{{\left(G*/\sin\delta \right)}_{\max}-{\left(G*/\sin\delta \right)}_{\mathrm{avg}}}{{\left(G*/\sin\delta \right)}_{\mathrm{avg}}}\times 100\%$$

(2) $$Separation\;index=\frac{{\left(Jnr\right)}_{\max}-{\left(Jnr\right)}_{\mathrm{avg}}}{{\left(Jnr\right)}_{\mathrm{avg}}}\times 100\%$$

(3) $$Separation\;index=\frac{{\left(\%rec\right)}_{\max}-{\left(\%rec\right)}_{\mathrm{avg}}}{{\left(\%rec\right)}_{\mathrm{avg}}}\times 100\%$$

## 3. Results and Discussion

### 3.1. Rheological Properties for Original Condition

#### 3.1.1. G*/sin δ

**Figure 6:**
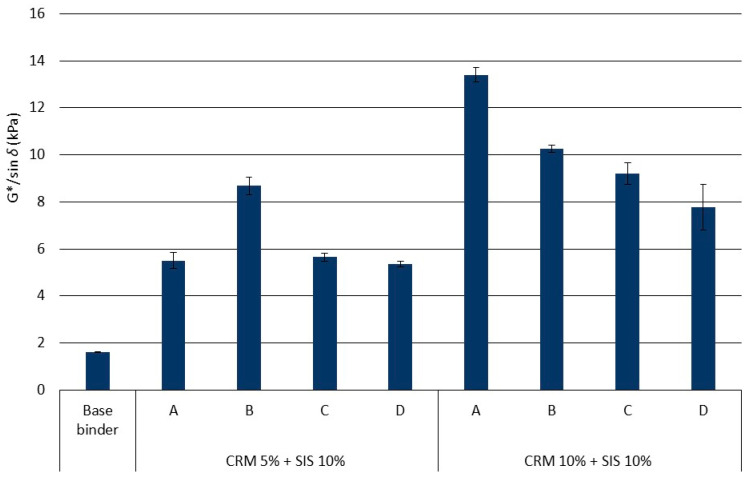
G*/sin *δ* of asphalt binders in each mixing method for the original condition.

The rheological properties of the binder were assessed by evaluating G*/sin *δ*. Overall, the addition of the modifier resulted in an increase in G*/sin *δ*, showing that as the CRM content increased, there was a clear tendency for G*/sin *δ* to increase (Figure 6). This outcome is attributed to the CRM’s absorption of the light oil in the binder, leading to a relative increase in asphaltene content and gelling of the CRM. In the SIS binder with 5% CRM, the results obtained from Method B showed a slight increase, which is thought to be due to the uneven dispersion of SIS at high temperatures, resulting in the partial reaggregation of SIS. This result affected the binder using 10% CRM, which shows an uneven trend and the lowest resulting gap among each mixing method. In each mixing method for CRM 10% + SIS 10% asphalt binder, G*/sin *δ* was relatively lower in groups C and D, which utilize the low-speed mixing approach for 6 h, compared to groups A and B. This is attributed to the suboptimal performance of SIS, a styrene block copolymer, due to polymer molecular chain bond cracking induced by the prolonged reaction at high temperatures. Consequently, it is evident that there are significant differences depending on the modifying method and duration, underscoring the need to consider various conditions to ensure optimal performance under field conditions (e.g., rpm for the modification, temperature, etc.).

#### 3.1.2. J_nr_ and % rec δ

**Figure 7:**
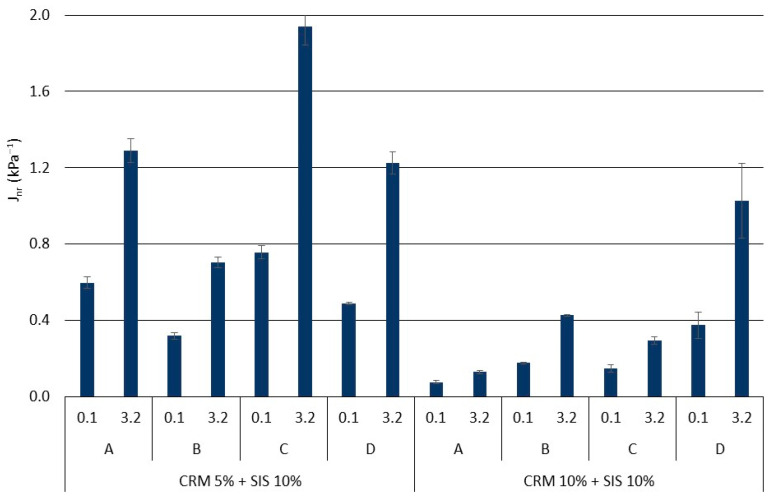
J_nr_ of asphalt binders in each mixing method for the original condition.

**Figure 8:**
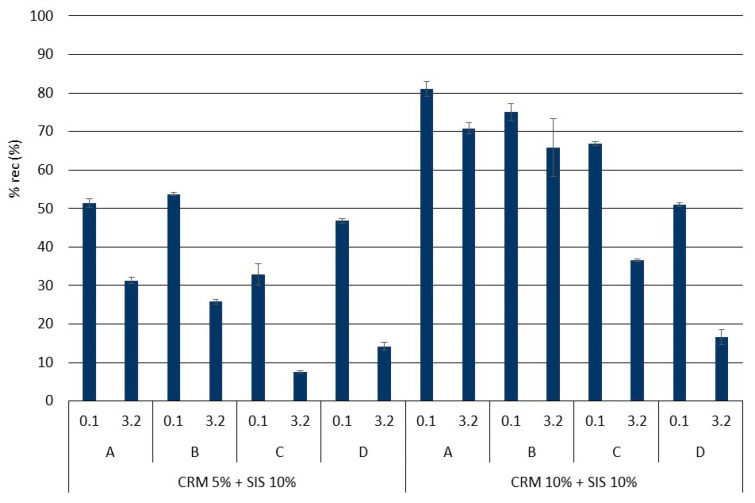
% rec of asphalt binders in each mixing method for the original condition.

A comprehensive analysis of rheological properties was conducted using J_nr_ and % rec, which employ a relatively higher load compared to the G*/sin *δ* evaluation method (Figure 7 and Figure 8). Overall, similar to the findings from the G*/sin *δ* evaluation, the J_nr_ value exhibited a decreasing trend with an increase in CRM content, while the % rec value showed an increasing trend. Notably, when applying a low load (0.1 kPa), the J_nr_ value tended to be lower and the % rec value tended to be higher, indicating a better elastic recovery of the binder under relatively lower load conditions compared to higher ones. Moreover, minimal deviation among results was observed between loads in the SIS asphalt binder with 10% CRM, suggesting that a higher CRM content renders the binder more elastic, enabling effective elastic recovery irrespective of the applied load magnitude. This trend was similarly reflected in the % rec results. Furthermore, a prolonged modification time (Methods C and D) led to a decline in the binder’s elastic recovery, mirroring the observations from the G*/sin *δ* evaluation. This phenomenon is attributed to the cracking of polymer chains in SIS due to a prolonged exposure to high temperature and reaction duration. Additionally, a notable decrease in elasticity was observed when applying a load of 3.2 kPa in Methods C and D of the group using long-term modification, indicating a heightened sensitivity in evaluating binder elasticity under relatively higher loads compared to G*/sin *δ* evaluation. These findings underscore the clearer depiction of reduced elasticity in the modified binder when subjected to prolonged modification at high temperature, as observed in this study.

### 3.2. Storage Stability Results

#### 3.2.1. SI Result Based on G*/sin δ

**Figure 9:**
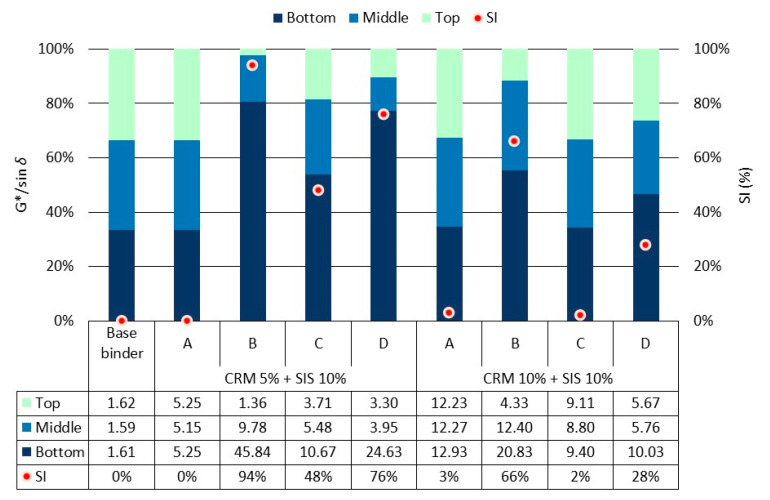
G*/sin *δ* of asphalt binders in each mixing method for top, middle, and bottom parts after conditioning.

The binder’s storage stability was assessed by calculating the SI utilizing the G*/sin *δ* value as a factor (Figure 9). In general, within the groups employing high-shear mixing methods (Groups A and C), there was a consistent distribution of G*/sin *δ* values from top to bottom, accompanied by a downward trend in SI values. It is considered that this phenomenon arises due to the uniform dispersion of the modifier facilitated by the high-shear mixer. Nevertheless, for the SIS binder employing 5% CRM in Method C, the SI was marginally higher than that of the SIS binder utilizing 10% CRM. This suggests that with prolonged modification time, the SIS binder that dispersed within the modified asphalt binder tends to re-agglomerate, potentially exerting a detrimental impact on asphalt binder performance, but in the result of the SIS binder using 10% CRM, a higher content of CRM hindered the reaggregation of SIS. On the other hand, the groups that chose the low-speed agitating method (B and D) showed high SI results. In the group utilizing Method B, it was observed that the G*/sin *δ* value exhibited a notably higher magnitude at the bottom. This is thought to occur due to the uneven dispersion of the SIS modifier at an elevated modifying temperature, leading to re-agglomeration and the subsequent settling of a higher concentration towards the bottom. Conversely, with the addition of a CRM up to 10%, certain SI values showed a tendency to decrease. This phenomenon is attributed to the increased CRM particles, which are considered to impede the entanglement of SIS at elevated temperatures. For Method D, the observed trend indicated lower SI values compared to Method B. This discrepancy can be attributed to the generally reduced G*/sin *δ* values resulting from prolonged modification at a high temperature, which induces degradation in the polymer molecular chain bonds of SIS. Consequently, it was observed that SIS, as a styrene block copolymer, exhibits significant sensitivity to both the modification method and temperature, meaning that it is imperative to identify the optimal modification conditions when employing it as a modifier.

#### 3.2.2. SI Result Based on J_nr_ and % Rec

**Figure 10:**
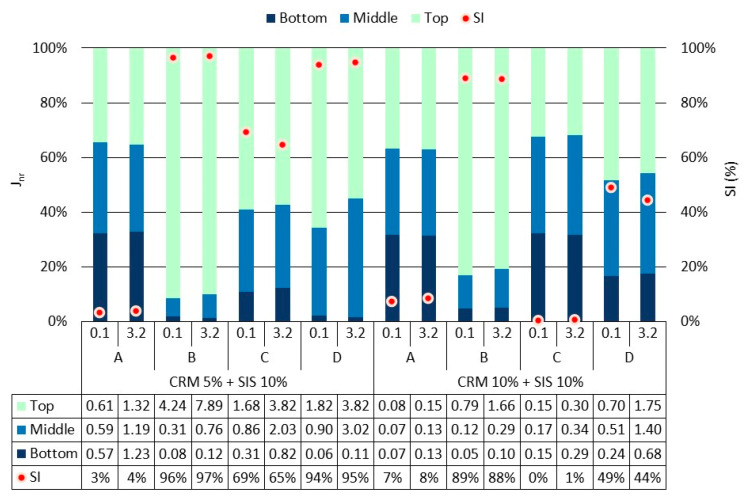
J_nr_ of asphalt binders in each mixing method for top, middle, and bottom parts after conditioning.

**Figure 11:**
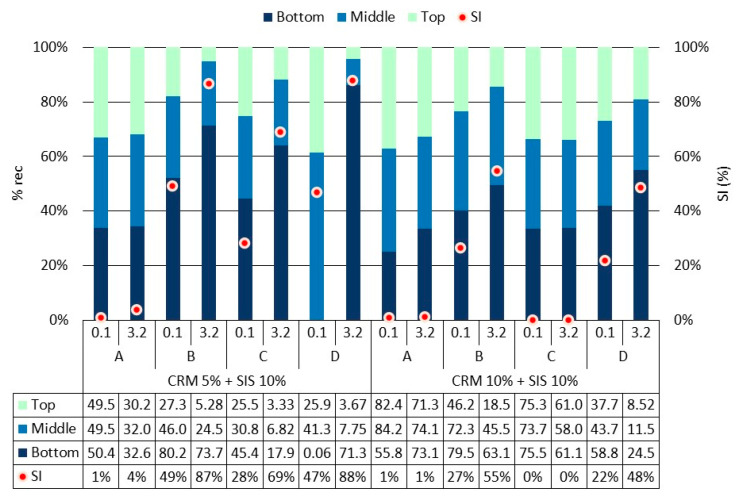
% rec of asphalt binders in each mixing method for top, middle, and bottom parts after conditioning.

Using the MSCR evaluation method, the storage stability of the binder was assessed by calculating the SI based on the J_nr_ and % rec values as factors (Figure 10 and Figure 11). The overall pattern observed in the SI results mirrored that of the SI results obtained from the G*/sin *δ* values. This indicates that the analysis of the modifier’s behavior based on the mixing method aligns almost consistently with the assessment of the G*/sin *δ* value. The SI values obtained using J_nr_ showed consistent results across both loading conditions without notable variations. This is considered to occur because the range of J_nr_ values is relatively narrow, typically less than 10 Kpa^−1^, and does not vary significantly when calculating the SI. Conversely, when computing % rec, it becomes apparent that the resulting value fluctuates depending on the applied load. This is attributed to the increased sensitivity of SI determination, as the result range spans from 0% to 100%, allowing for more discernible variations in % rec values. Specifically, with the application of a 3.2 kPa load, the % rec values were notably higher for all binders, also showing that some results of certain mixing methods were nearly double compared to when a 0.1 kPa load was applied. These outcomes are attributed to the enhanced clarity in measuring the binder’s elasticity when subjecting it to a relatively higher load. Therefore, when assessing the SI of the binder using the MSCR evaluation method, it is anticipated that the storage stability of the binder will be scrutinized more sensitively by % rec applied with a 3.2 kPa load; so, it can be utilized as a factor.

### 3.3. Comparative Analysis of the SI Based on Each Factor in Mixing Methods

**Figure 12:**
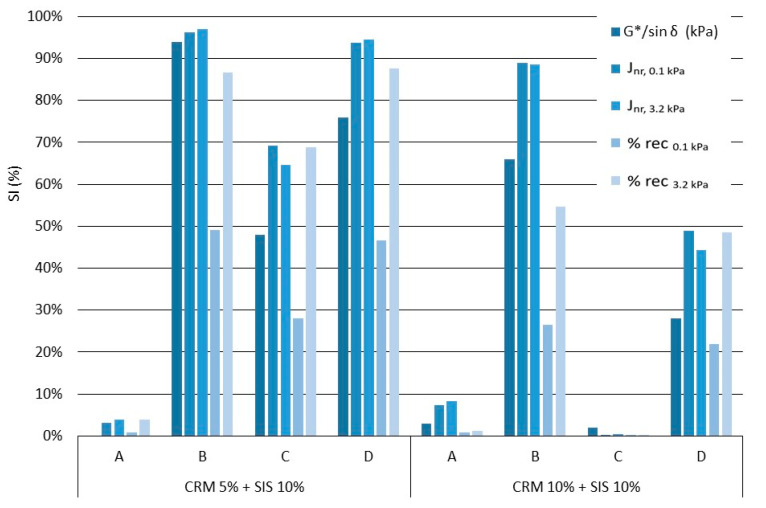
SI for each mixing method.

To evaluate the storage stability of the asphalt binder, the ASTM D7173 guidelines mention that the selection of a test for this purpose will vary depending on the polymer modification system under evaluation and the specific information sought by the user. The DSR test [40] is widely utilized for this purpose, while the MSCR Test [41] serves as an alternative test option. Therefore, to derive factors for assessing storage stability, namely, calculating SI, individual test methods were chosen (Figure 12). In general, variations in SI results were observed depending on the chosen mixing method; lower SI results were evident when employing the high-shear mixing approach. This is considered to occur due to differences in the reaction of the modifier, influenced by factors such as the modification method, temperature, and duration, as mentioned in the storage stability findings. Moreover, disparities were observed in the SI results across various test factors within each mixing method. Specifically, the evaluation of the MSCR test displayed variations depending on the applied load, with higher SI results observed when a high load was applied. This is attributed to the heightened sensitivity of the high load to the elastic behavior of the binder.

However, when evaluating J_nr_ as a factor, no significant difference was observed across varying loads. This is likely because the result of the factor is less than 10 kPa^−1^, indicating a limited range that does not allow for the sensitive measurement of the binder’s SI. Therefore, for a more precise evaluation of SI, it is deemed preferable to utilize % rec as a factor, given its broader range of values spanning from 0 to 100%, rather than deriving SI based on J_nr_ evaluations. Consequently, while the ASTM D7173 guidelines permit the assessment of binder storage stability through DSR and MSCR tests, achieving consistent results regarding the extent of separation of the modified binder between G*/sin δ, J_nr_, and % rec parameter values has proven challenging.

### 3.4. Statistical Analysis among SI Results

A statistical analysis was conducted utilizing the IBM statistical package for the social sciences (SPSS) to carry out an analysis of variance (ANOVA) and Fisher’s least significant difference (LSD) comparison with a significance level of α = 0.05. The statistical design was based on evaluating G*/sin *δ*, J_nr_, and % rec in each mixing method. An ANOVA was initially performed to ascertain the significant differences among the sample means. In general, the SI results exhibited statistically significant differences depending on the mixing method in the SIS asphalt binder with 5% CRM added. Specifically, in Method A, it was established that all SI results were statistically similar. However, significant differences were observed between the SI results of different mixing methods (B, C, D), as shown in Table 4. Similarly, in the SIS asphalt binder with 10% CRM added, comparable results were found among the SI results in each method within the high-shear mixing group (Table 5). Significant differences were noted among the SI results in the low-speed agitating group, except for Method C. Consequently, notable distinctions were generally observed among mixing methods and factors used to evaluate SI. In summary, opting for the high-shear mixing method yielded a consistent pattern of SI results for each parameter value. Nonetheless, this uniformity stemmed from only using a similar and lower SI by factors to calculate the SI results. This highlights the need for comprehensive consideration between test methods and evaluation factors to analyze a broad range of SI results effectively.

## 4. Summary and Conclusions

The storage stability of asphalt binders was examined by utilizing G*/sin *δ*, J_nr_, and % rec as factors to assess a 10% SIS binder with the inclusion of a 5% and 10% CRM content, both before and after conditioning using four mixing methods. The findings of this investigation led to the following conclusions.

(1) Overall, the addition of the modifier increased the G*/sin *δ* values, correlating with the CRM increase. Additionally, different mixing methods and modifier contents resulted in varied effects on G*/sin *δ*. Groups with a low-speed mixing for 6 h exhibited lower G*/sin *δ* values, indicating a suboptimal SIS performance based on the chain cracking of SIS. These results emphasize the importance of considering modifying the methods and duration for optimal binder performance.
(2) The rheological properties using J_nr_ and % rec revealed consistent trends similar to G*/sin *δ* evaluation, indicating a decreasing J_nr_ value and increasing % rec value with a higher CRM content. In general, under a low load (0.1 kPa), lower J_nr_ and higher % rec values suggest improved elastic recovery than under a load of 3.2 kPa. Moreover, using higher loads (3.2 kPa) and a long-term modification method showed a significant decrease in elasticity, highlighting increased sensitivity compared to the G*/sin *δ* evaluation. These findings emphasize the clearer understanding of reduced elasticity in modified binder under prolonged high-temperature modification.
(3) The storage stability of the binder was evaluated using G*/sin *δ*, revealing consistent trends in the high-shear mixing groups (A and C) with a downward SI trend, indicating uniform modifier dispersion. The low-speed mixing groups (B and D) showed an elevated SI, possibly due to the uneven SIS dispersion causing re-agglomeration. Method D exhibited a lower SI compared to Method B, which is attributed to the increased G*/sin *δ* values of bottom part from prolonged high-temperature modification, indicating SIS sensitivity to modification conditions. Thus, SIS exhibits significant sensitivity to both the modification method and temperature during asphalt binder modification.
(4) The storage stability of the binder was evaluated using the MSCR method, which involved calculating the SI based on J_nr_ and % rec values. J_nr_-based SI values showed consistency across loading conditions due to the narrow range of J_nr_ values, whereas the % rec-based SI exhibited fluctuation, attributed to its wider range. Particularly, at a 3.2 kPa load, the % rec values significantly increased, indicating enhanced elasticity measurement under higher loads. Thus, % rec with a 3.2 kPa load is effective for the sensitive evaluation of binder storage stability using the MSCR method.
(5) J_nr_, a factor in evaluating the storage stability, shows no significant difference across varying loads, likely due to its limited range (<10 kPa^−1^), hindering sensitive SI measurement. Thus, % rec is preferred for precise SI evaluation, offering a broader range (0 to 100%). In addition, despite the ASTM D7173 guidelines permitting DSR and MSCR tests for binder assessment, achieving consistent results regarding modified binder separation between G*/sin *δ*, J_nr_, and % rec parameters remains challenging. These results also unveiled variances through statistical analysis.
(6) This research exclusively employed a CRM and SIS; hence, the investigation into the behavior of other modifiers is required. In the future, a comprehensive analysis will likely necessitate the inclusion of diverse modifiers and a deeper examination of their direct influence on asphalt mixtures. Furthermore, to assess the degree of separation between the binder and the modifier, along with conducting a phase separation analysis of the binder, it is crucial to consider suitable evaluation factors. It is anticipated that endeavors will be required to establish an optimal evaluation method.

## Figures and Tables

**Table 1:** Properties of base asphalt binder (PG 64-22).

Aging States	Test Properties	Test Result	Minimum Specification
Unaged binder	Viscosity @ 135 °C	527 cP	<3000 cP
	G*/sin δ @ 64 °C	1.373 kPa	>1.00 kPa
RTFO aged residual	G*/sin δ @ 64 °C	3.751 kPa	>2.2 kPa
RTFO + PAV Aged residual	G*sin δ @ 25 °C	4245 kPa	<5000 kPa
	Stiffness @ −12 °C	214 MPa	<300 MPa
	m-value @ −12 °C	0.318	> 0.3

**Table 2:** Passing rate of Crumb rubber modifier adopted in this study.

Sieve Number (μm)	Passing Rate (%)
30 (600)	100
50 (300)	57.7
100 (150)	14.2
200 (75)	0.0

**Table 3:** Properties of SIS.

Polymer Structure	Linear
Styrene, wt %	15
Diblock, wt %	18
Melt flow, g/10min (200 °C/5kg)	11
Solution viscosity, cps	1240
Ash, wt %	0.3
Volatiles, wt %	0.2
Specific gravity	0.92
Tensile strength, psi (MPa)	3600 (25)
Elongation, %	1250
Hardness, shore A	33

**Table 4:** Statistical analysis of CRM5% + SIS10% asphalt binder for SI based on testing and mixing methods (α = 0.05).

			CRM5% + SIS10%																			
			G*/sin *δ* (kPa)				J_nr_ (0.1 kPa^−1^)				J_nr_ (3.2 kPa^−1^)				% rec (0.1 kPa^−1^)				% rec (3.2 kPa^−1^)			
			A	B	C	D	A	B	C	D	A	B	C	D	A	B	C	D	A	B	C	D
CRM5% + SIS10%	G*/sin *δ* (kPa)	A	-	S	S	S	N	S	S	S	N	S	S	S	N	S	S	S	N	S	S	S
		B		-	S	S	S	N	S	N	S	N	S	N	S	S	S	S	S	S	S	S
		C			-	S	S	S	S	S	S	S	S	S	S	N	S	N	S	S	S	S
		D				-	S	S	S	S	S	S	S	S	S	S	S	S	S	S	S	S
	J_nr_ (0.1 kPa^−1^)	A					-	S	S	S	N	S	S	S	N	S	S	S	N	S	S	S
		B						-	S	N	S	N	S	N	S	S	S	S	S	S	S	S
		C							-	S	S	S	S	S	S	S	S	S	S	S	N	S
		D								-	S	N	S	N	S	S	S	S	S	S	S	S
	J_nr_ (3.2 kPa^−1^)	A									-	S	S	S	N	S	S	S	N	S	S	S
		B										-	S	N	S	S	S	S	S	S	S	S
		C											-	S	S	S	S	S	S	S	N	S
		D												-	S	S	S	S	S	S	S	S
	% rec (0.1 kPa^−1^)	A													-	S	S	S	N	S	S	S
		B														-	S	N	S	S	S	S
		C															-	S	S	S	S	S
		D																-	S	S	S	S
	% rec (3.2 kPa^−1^)	A																	-	S	S	S
		B																		-	S	N
		C																			-	S
		D																				-

N: non-significant; S: significant.

**Table 5:** Statistical analysis of CRM10% + SIS10% asphalt binder for SI based on testing and mixing methods (α = 0.05).

			CRM10% + SIS10%																			
			G*/sin *δ* (kPa)				J_nr_ (0.1 kPa^−1^)				J_nr_ (3.2 kPa^−1^)				% rec (0.1 kPa^−1^)				% rec (3.2 kPa^−1^)			
			A	B	C	D	A	B	C	D	A	B	C	D	A	B	C	D	A	B	C	D
CRM10% + SIS10%	G*/sin *δ* (kPa)	A	-	S	N	S	N	S	N	S	N	S	N	S	N	S	N	S	N	S	N	S
		B		-	S	S	S	S	S	S	S	S	S	S	S	S	S	S	S	S	S	S
		C			-	S	N	S	N	S	N	S	N	S	N	S	N	S	N	S	N	S
		D				-	S	S	S	S	S	S	S	S	S	N	S	N	S	S	S	S
	J_nr_ (0.1 kPa^−1^)	A					-	S	N	S	N	S	N	S	N	S	S	S	N	S	S	S
		B						-	S	S	S	N	S	S	S	S	S	S	S	S	S	S
		C							-	S	S	S	N	S	N	S	N	S	N	S	N	S
		D								-	S	S	S	N	S	S	S	S	S	N	S	N
	J_nr_ (3.2 kPa^−1^)	A									-	S	N	S	N	S	S	S	N	S	S	S
		B										-	S	S	S	S	S	S	S	S	S	S
		C											-	S	N	S	N	S	N	S	N	S
		D												-	S	S	S	S	S	S	S	N
	% rec (0.1 kPa^−1^)	A													-	S	N	S	N	S	N	S
		B														-	S	N	S	S	S	S
		C															-	S	N	S	N	S
		D																-	S	S	S	S
	% rec (3.2 kPa^−1^)	A																	-	S	N	S
		B																		-	S	N
		C																			-	S
		D																				-

N: non-significant; S: significant.

## Data Availability

Data are contained within the article.
